# Quantum Characteristics of a Nanomechanical Resonator Coupled to a Superconducting LC Resonator in Quantum Computing Systems

**DOI:** 10.3390/nano9010020

**Published:** 2018-12-24

**Authors:** Jeong Ryeol Choi, Sanghyun Ju

**Affiliations:** Department of Physics, Kyonggi University, Yeongtong-gu, Suwon, Gyeonggi-do 16227, Korea; shju@kyonggi.ac.kr

**Keywords:** nanomechanical resonator, superconducting resonator, wave function, unitary transformation, Hamiltonian, probability density, adiabatic condition, quantum solution

## Abstract

The mechanical and quantum properties of a nanomechanical resonator can be improved by connecting it to a superconducting resonator in a way that the resonator exhibits new phenomena that are possibly available to novel quantum technologies. The quantum characteristics of a nanomechanical resonator coupled to a superconducting resonator have been investigated on the basis of rigorous quantum solutions of the combined system. The solutions of the Schrödinger equation for the coupled system have been derived using the unitary transformation approach. The analytic formula of the wave functions has been obtained by applying the adiabatic condition for time evolution of the coupling parameter. The behavior of the quantum wave functions has been analyzed for several different values of parameters. The probability densities depicted in the plane of the two resonator coordinates are distorted and rotated due to the coupling between the resonators. In addition, we have shown that there are squeezing effects in the wave packet along one of the two resonator coordinates or along both the two depending on the magnitude of several parameters, such as mass, inductance, and angular frequencies.

## 1. Introduction

A rapidly developing field in nano-based science and technology is optomechanics which deals with the interaction of light with a mechanical motion [1]. Especially, optomechanics combined with nanomechanical resonators can be practically applied to a broad scientific domain such as quantum information processing [2], biological sensing [3], wave detections [4], measurements of mechanical displacement [5], and quantum metrologies [6]. Such optomechanics developed in the quantum regime with low phonon occupation states will play a major role in future-oriented quantum technologies with nanodevices.

The investigation of nanodevices regarding their application in quantum information science, including quantum computing, is a promising research topic. In particular, research into nanomechanical resonators in which the parameters are dependent on time is quite necessary for the advancement of the quantum information technology [7,8,9,10,11]. Now, it is possible to design quantum computing devices with a reliable architecture for multi-qubit operations in the GHz-frequency range by coupling mechanical resonators to Josephson phase qubits [12]. Onchip-integrated hybrid systems, i.e., mechanical resonators combined with phase qubits which are composed of Josephson-junction superconducting circuits, can be used as a compact quantum information storage with a high quality factor [13]. Recent advances in nanotechnology in the past decade enabled the fabrication of nanomechanical resonators, of which quality factors are high in the desired frequency ranges.

By connecting nanomechanical resonators to the superconducting resonators, it is possible to enhance the properties of nanomechanical resonators. Thanks to this, many relevant experiments have been developed and renewed, such as, for example, preparing quantum ground state in resonators [10], a frequency up/down conversion [11], and maintaining longer coherence times [14]. In particular, coherent feedback control of the nanoresonators can be used for cooling them to the zero-point temperature. The cooling of a mechanical system to the ground-state has already been achieved in several laboratories [15,16].

As the size of the resonator reaches below a critical value that is the limiting one from a quantum mechanical point of view, there emerge distinguishing quantum features that are totally absent in the classical world [5]. Quantum behaviors of a nanomechanical resonator were observed through its sideband laser-cooling over the quantum ground state [17]. A deeper understanding of quantum characteristics of nanomechanical resonators, where the device-size is within the Heisenberg uncertainty principle limit, is crucial for utilizing them in quantum computing systems. We investigate quantum properties of a nanomechanical resonator coupled to a superconducting resonator. This is important for providing theoretical knowledge as background information for manipulating quantum computing processes. Furthermore, this research may help to achieve robust quantum computations on the basis of the restriction of the decoherence and noise.

## 2. Materials and Methods

Cooling of nanomechanical resonators can be achieved by the methods of sideband cooling or measurement-based feedback cooling [18]. The former is classified as coherent feedback controlling for a nanomechanical system, that can be carried out by coupling it to an auxiliary one such as an optical cavity and a superconducting oscillator, whereas the systems in the letter case are controlled by means of the feedback technique with the data which are continuously measured from homodyne detection. In this work, we consider sideband cooling using a high-frequency superconducting LC oscillator as an auxiliary mode in order to control the motion of the nanomechanical resonator. For more details of the nanomechanical-resonator design and the related mechanics, refer to Refs. [10,11].

To see quantum features of the system, we first need to derive relevant quantum solutions in a rigorous way. We introduce the Hamiltonian for describing the quantum dynamics of the nanomechanical resonator coupled to the superconducting resonator. The Hamiltonian involves a coupling term which is associated with the coupling of the two resonators. Due to not only such a coupling term but the time-dependence of the parameters as well, it may be not an easy task to solve the Schrödinger equation on the basis of the conventional separation of variables method. For this reason, we will derive quantum solutions by making use of another method which is the unitary transformation method [7,8,19].

It may be convenient that we unfold quantum theory after mathematically transforming the system described by a time-dependent Hamiltonian into a simple one that can be easily managed. By introducing a unitary operator, we will transform the original system to a system composed of two decoupled harmonic oscillators of which quantum solutions are well known. By inversely transforming the quantum solutions associated to the transformed system, it is possible to obtain the complete quantum solutions in the (original) system. This is the main strategy used in this work for deriving quantum solutions of the system whose Hamiltonian is a fairly complicated form. We will analyze quantum properties of the system in detail on the basis of the quantum solutions evaluated in such a way.

## 3. Results and Discussion

### 3.1. Hamiltonian and the Unitary Transformation

Superconducting systems can be used to improve the properties of other quantum systems by connecting them to the target systems [20]. From there, we can explore new phenomena which could possibly bring about the deveopment of novel quantum technologies for quantum information processing. For instance, a sideband cooling of a nanoresonator is possible through its modulation via coupling it to a superconducting LC oscillator. In this case, we can regard the nanoresonator as the target device and the superconducting LC resonator as an auxiliary one.

The Hamiltonian that describes the nanomechanical resonator combined with the superconducting LC resonator is given by [10,11]

(1) $$\hat{H}=\hbar \omega \left({\hat{a}}^{†}\hat{a}+1/2\right)+\hbar \Omega \left({\hat{b}}^{†}\hat{b}+1/2\right)+\hbar \lambda \left(t\right)\left(\hat{a}+{\hat{a}}^{†}\right)\left(\hat{b}+{\hat{b}}^{†}\right),$$

where $\hat{a}$ and $\hat{b}$ are annihilation operators in the nanomechanical resonator and the superconducting resonator, respectively, and ω is the frequency of the nanoresonator, while Ω is the frequency of the superconducting resonator. Here, we assume that the parameter λ(t) is a slowly varying function so that we can apply the adiabatic theorem. Although we are interested in the quantum description of the nanomechanical resonator coupled to the superconducting resonator with Equation (1), the Hamiltonian in Equation (1) can also be used for other purposes such as the gauge field theory of two superconducting resonators which are tunably coupled to each other [21] and quantum simulation of bosonic modes utilizing superconducting circuits [22].

Because the operators $\hat{a}$ and $\hat{b}$ are represented in the form

(2) $$\begin{array}{ccc} \hat{a} & = & \sqrt{\frac{m\omega }{2\hbar }}\hat{x}+\frac{i{\hat{p}}_{x}}{\sqrt{2\hbar m\omega }}, \end{array}$$

(3) $$\begin{array}{ccc} \hat{b} & = & \sqrt{\frac{L\Omega }{2\hbar }}\hat{q}+\frac{i{\hat{p}}_{q}}{\sqrt{2\hbar L\Omega }}, \end{array}$$

with ${\hat{p}}_{x}=-i\hbar \partial /\partial x$ and ${\hat{p}}_{q}=-i\hbar \partial /\partial q$, Equation (1) can be rewritten as

(4) $$\hat{H}=\frac{{\hat{p}}_{x}^{2}}{2m}+\frac{1}{2}m\omega^{2}{\hat{x}}^{2}+\frac{{\hat{p}}_{q}^{2}}{2L}+\frac{1}{2}L\Omega^{2}{\hat{q}}^{2}+2\lambda \left(t\right)\sqrt{mL\omega \Omega }\hat{x}\hat{q}.$$

Due to the coupling term (the last term) in the above equation, it may be somewhat difficult to treat this system from a quantum mechanical point of view. In order to overcome this, we will mathematically decouple the two sub-systems by means of the unitary transformation method [7,8,19]. For this purpose, we introduce a unitary operator of the form

(5) $$\begin{array}{ccc} \hat{U} & = & \exp\left[\frac{i}{4\hbar }\left({\hat{p}}_{x}\hat{x}+\hat{x}{\hat{p}}_{x}\right)\ln\left(\sqrt{m/L}\right)\right]\\ & & \times \exp\left[\frac{i}{4\hbar }\left({\hat{p}}_{q}\hat{q}+\hat{q}{\hat{p}}_{q}\right)\ln\left(\sqrt{L/m}\right)\right]\\ & & \times \exp\left[-\frac{i\theta (t)}{\hbar }\left({\hat{p}}_{x}\hat{q}-{\hat{p}}_{q}\hat{x}\right)\right], \end{array}$$

where

(6) $$\theta \left(t\right)=\frac{1}{2}\tan^{-1}\left(\frac{4\lambda \left(t\right)\sqrt{\omega \Omega }}{\Omega^{2}-\omega^{2}}\right).$$

By transforming $\hat{H}$ in Equation (4) using this operator:

(7) $${\hat{H}}^{\prime }={\hat{U}}^{-1}\hat{H}\hat{U}-i\hbar {\hat{U}}^{-1}\frac{\partial \hat{U}}{\partial t},$$

we have the Hamiltonian in the transformed system as

(8) $${\hat{H}}^{\prime }=\frac{{\hat{p}}_{x}^{2}+{\hat{p}}_{q}^{2}}{2\sqrt{mL}}+\frac{1}{2}\sqrt{mL}w_{1}^{2}\left(t\right){\hat{x}}^{2}+\frac{1}{2}\sqrt{mL}w_{2}^{2}\left(t\right){\hat{q}}^{2}+\dot{\lambda }\left(t\right)\beta \left(t\right)\left({\hat{p}}_{x}\hat{q}-{\hat{p}}_{q}\hat{x}\right),$$

where

(9) $$\begin{array}{ccc} w_{1}^{2}\left(t\right) & = & \omega^{2}\cos^{2}\theta \left(t\right)+\Omega^{2}\sin^{2}\theta \left(t\right)-4\lambda \left(t\right)\sqrt{\omega \Omega }\cos\theta \left(t\right)\sin\theta \left(t\right), \end{array}$$

(10) $$\begin{array}{ccc} w_{2}^{2}\left(t\right) & = & \omega^{2}\sin^{2}\theta \left(t\right)+\Omega^{2}\cos^{2}\theta \left(t\right)+4\lambda \left(t\right)\sqrt{\omega \Omega }\cos\theta \left(t\right)\sin\theta \left(t\right), \end{array}$$

(11) $$\begin{array}{ccc} \beta (t) & = & -\frac{2\sqrt{\omega \Omega }}{\Omega^{2}-\omega^{2}+16\omega \Omega \lambda^{2}\left(t\right)/\left(\Omega^{2}-\omega^{2}\right)}. \end{array}$$

According to the adiabatic condition which is that λ(t) is a sufficiently slowly time-varying function, we can neglect the last term in Equation (8), leading to

(12) $${\hat{H}}^{\prime }≃\frac{{\hat{p}}_{x}^{2}+{\hat{p}}_{q}^{2}}{2\mu }+\frac{1}{2}\mu w_{1}^{2}\left(t\right){\hat{x}}^{2}+\frac{1}{2}\mu w_{2}^{2}\left(t\right){\hat{q}}^{2},$$

where $\mu =\sqrt{mL}$. Thus, the two sub-systems are decoupled as can be confirmed from the above Hamiltonian. The quantum treatment of the system relying on the transformed Hamiltonian, Equation (12), may be much simpler than that relying on the original Hamiltonian given in Equation (4). Two independent classical equations of motion that correspond to ${\hat{H}}^{\prime }$ are given by

(13) $$\frac{d^{2}x}{dt^{2}}+w_{1}^{2}\left(t\right)x=0,$$

(14) $$\frac{d^{2}q}{dt^{2}}+w_{2}^{2}\left(t\right)q=0.$$

In the next section, we will derive the quantum wave solutions associated to the Hamiltonian given in Equation (12). By using the unitary relation between the wave functions in the transformed system and those in the original system, the quantum wave solutions in the original system will be obtained and analyzed.

### 3.2. Quantum Wave Solutions

From the knowledge of the formulae of quantum wave functions in the transformed system, we can obtain the wave functions in the original system because the two systems are connected by a unitary operator. If we denote the wave functions in the transformed system associated with Equations (13) and (14) as $\psi_{n}^{\prime }\left(x,t\right)$ and ${\tilde{\psi }}_{l}^{\prime }\left(q,t\right)$, respectively, they can be divided into kernel and phase parts such that

(15) $$\begin{array}{ccc} \psi_{n}^{\prime }\left(x,t\right) & = & \phi_{n}^{\prime }\left(x,t\right)\exp\left[i\alpha_{n}\left(t\right)\right], \end{array}$$

(16) $$\begin{array}{ccc} {\tilde{\psi }}_{l}^{\prime }\left(q,t\right) & = & {\tilde{\phi }}_{l}^{\prime }\left(q,t\right)\exp\left[i{\tilde{\alpha }}_{l}\left(t\right)\right], \end{array}$$

where $\alpha_{n}\left(t\right)$ and ${\tilde{\alpha }}_{l}\left(t\right)$ are time-dependent phases.

Let us write the corresponding Schrödinger equation as

(17) $$i\hbar \frac{\partial \Psi_{n,l}^{\prime }\left(x,q,t\right)}{\partial t}={\hat{H}}^{\prime }\Psi_{n,l}^{\prime }\left(x,q,t\right),$$

where $\Psi_{n,l}^{\prime }\left(x,q,t\right)$ are wave functions in the transformed system, that are of the form

(18) $$\Psi_{n,l}^{\prime }\left(x,q,t\right)=\psi_{n}^{\prime }\left(x,t\right){\tilde{\psi }}_{l}^{\prime }\left(q,t\right).$$

Because the transformed system is composed of the two decoupled harmonic oscillators, we can easily identify the corresponding quantum solutions, Equation (18). Then, the wave functions $\Psi_{n,l}\left(x,q,t\right)$ in the original system are obtained from such solutions through the use of the unitary relation $\Psi_{n,l}\left(x,q,t\right)=\hat{U}\Psi_{n,l}^{\prime }\left(x,q,t\right)$. According to this, the wave functions in the original system are represented as

(19) $$\Psi_{n,l}\left(x,q,t\right)=\psi_{n}\left(x,q,t\right){\tilde{\psi }}_{l}\left(x,q,t\right),$$

where

(20) $$\begin{array}{ccc} \psi_{n}\left(x,q,t\right) & = & \phi_{n}\left(x,q,t\right)\exp\left[i\alpha_{n}\left(t\right)\right], \end{array}$$

(21) $$\begin{array}{ccc} {\tilde{\psi }}_{l}\left(x,q,t\right) & = & {\tilde{\phi }}_{l}\left(x,q,t\right)\exp\left[i{\tilde{\alpha }}_{l}\left(t\right)\right], \end{array}$$

while

(22) $$\phi_{n}\left(x,q,t\right)=\hat{U}\phi_{n}^{\prime }\left(x,t\right),$$

(23) $${\tilde{\phi }}_{l}\left(x,q,t\right)=\hat{U}{\tilde{\phi }}_{l}^{\prime }\left(q,t\right).$$

**Figure 1:**
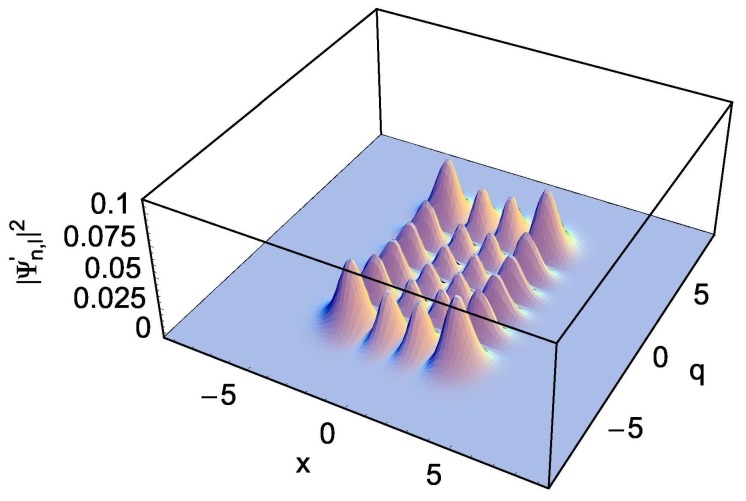
The probability density, $|\Psi_{n,l}^{\prime }{\left(x,q,t\right)|}^{2}$, in the transformed system with the choice of (*m*, *L*) = (1.0, 1.0) under the limit $\lambda \left(t\right)=\lambda_{0}$. This is associated to the wave functions, Equation (18) with Equations (15), (16), and (24)–(27). We used (*n*, *l*) = (3, 5), (ω, Ω) = (0.5, 0.49), ℏ=1, and $\lambda_{0}=0.1$.

Let us now further see for the case that the coupling parameter is a positive real constant, $\lambda \left(t\right)=\lambda_{0}$. In this case, $w_{i}$ (i=1,2) and θ become constants. As a consequence, the corresponding quantum solutions are easily identified to be

(24) $$\begin{array}{ccc} \alpha_{n}\left(t\right) & = & -\left(n+1/2\right)w_{1}t, \end{array}$$

(25) $$\begin{array}{ccc} {\tilde{\alpha }}_{l}\left(t\right) & = & -\left(l+1/2\right)w_{2}t, \end{array}$$

(26) $$\begin{array}{ccc} \phi_{n}^{\prime }\left(x\right) & = & {\left(\frac{\sqrt{k_{1}/\pi }}{2^{n}n!}\right)}^{1/2}H_{n}\left(\sqrt{k_{1}}x\right)\exp\left[-k_{1}x^{2}/2\right], \end{array}$$

(27) $$\begin{array}{ccc} {\tilde{\phi }}_{l}^{\prime }\left(q\right) & = & {\left(\frac{\sqrt{k_{2}/\pi }}{2^{l}l!}\right)}^{1/2}H_{l}\left(\sqrt{k_{2}}q\right)\exp\left[-k_{2}q^{2}/2\right], \end{array}$$

where $k_{i}=\mu w_{i}/\hbar$, and $H_{n(l)}$ are Hermite polynomials. The probability density, $|\Psi_{n,l}^{\prime }{\left(x,q,t\right)|}^{2}$, in the transformed system is illustrated in Figure 1 with the choice of (*n*, *l*) = (3, 5) under the limit that the coupling parameter is constant. This density is not deformed because the Hamiltonian of the transformed system does not involve the coupling term.

According to the relations, Equations (22) and (23), the eigenfunctions in the original system can be derived to be

(28) $$\begin{array}{ccc} \phi_{n}\left(x,q,t\right) & = & {\left(\frac{\sqrt{k_{1}/\pi }}{2^{n}n!}\right)}^{1/2}H_{n}\left(\sqrt{k_{1}}Q_{1}\right)\exp\left[-k_{1}Q_{1}^{2}/2\right], \end{array}$$

(29) $$\begin{array}{ccc} {\tilde{\phi }}_{l}\left(x,q,t\right) & = & {\left(\frac{\sqrt{k_{2}/\pi }}{2^{l}l!}\right)}^{1/2}H_{l}\left(\sqrt{k_{2}}Q_{2}\right)\exp\left[-k_{2}Q_{2}^{2}/2\right], \end{array}$$

where

(30) $$\begin{array}{ccc} Q_{1} & = & {\left(\frac{m}{L}\right)}^{1/4}\cos\theta \left(t\right)x-{\left(\frac{L}{m}\right)}^{1/4}\sin\theta \left(t\right)q, \end{array}$$

(31) $$\begin{array}{ccc} Q_{2} & = & {\left(\frac{m}{L}\right)}^{1/4}\sin\theta \left(t\right)x+{\left(\frac{L}{m}\right)}^{1/4}\cos\theta \left(t\right)q. \end{array}$$

**Figure 2:**
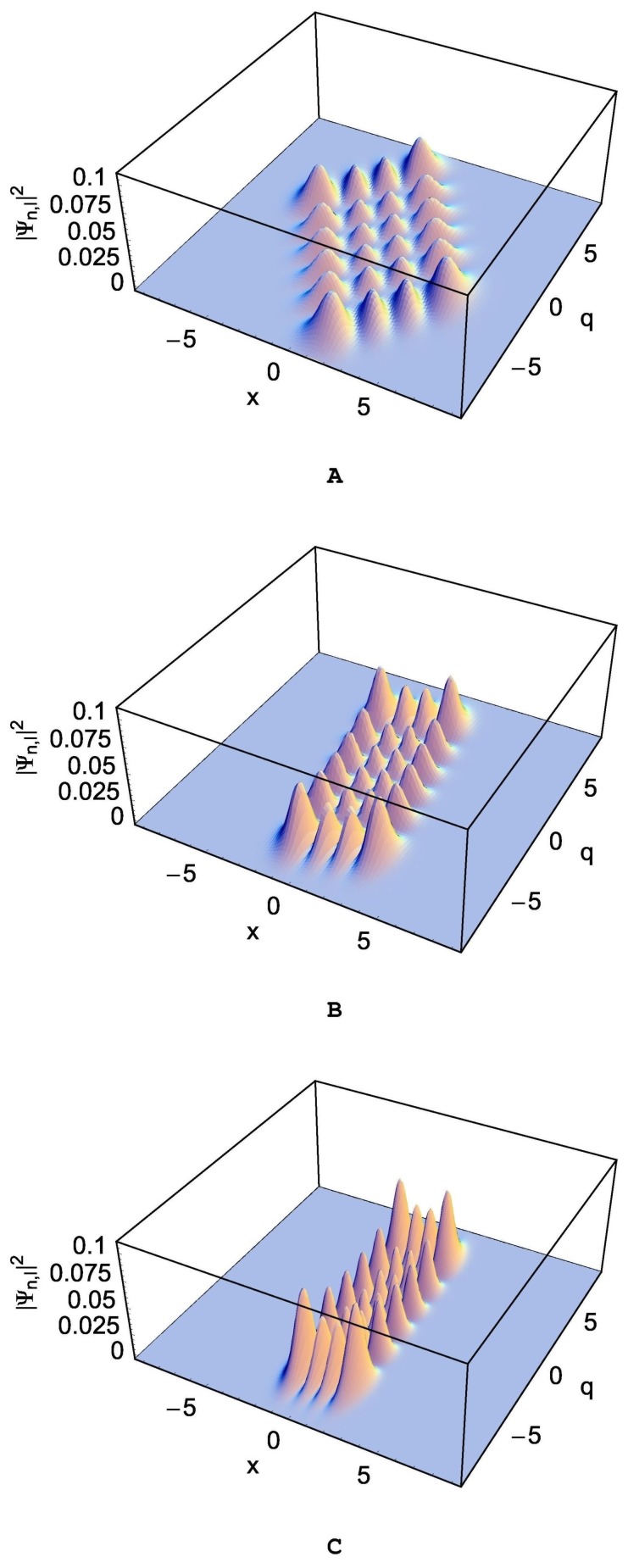
The probability density $|\Psi_{n,l}{\left(x,q,t\right)|}^{2}$ in the original system for several different values of ω with the choice of (*m*, *L*) = (1.0, 0.5) under the limit $\lambda \left(t\right)=\lambda_{0}$. This is associated to the wave functions given in Equation (19) with Equations (20), (21), (24), (25), (28), and (29). The values of (ω, Ω) are (0.5, 0.49) for (**A**), (1.0, 0.49) for (**B**), and (2.0, 0.49) for (**C**). We used (*n*, *l*) = (3, 5), ℏ=1, and $\lambda_{0}=0.1$.

**Figure 3:**
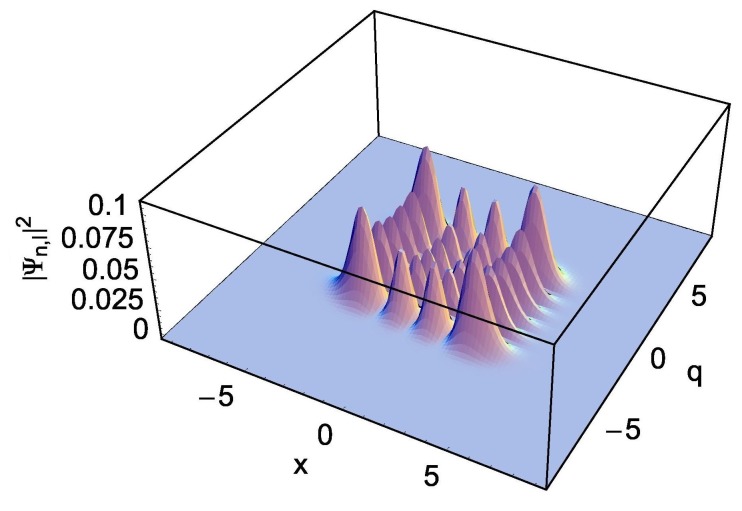
This figure is the same as Figure 2C, but for a different choice of angular frequencies. The values of (ω, Ω) are (0.49, 2.0).

**Figure 4:**
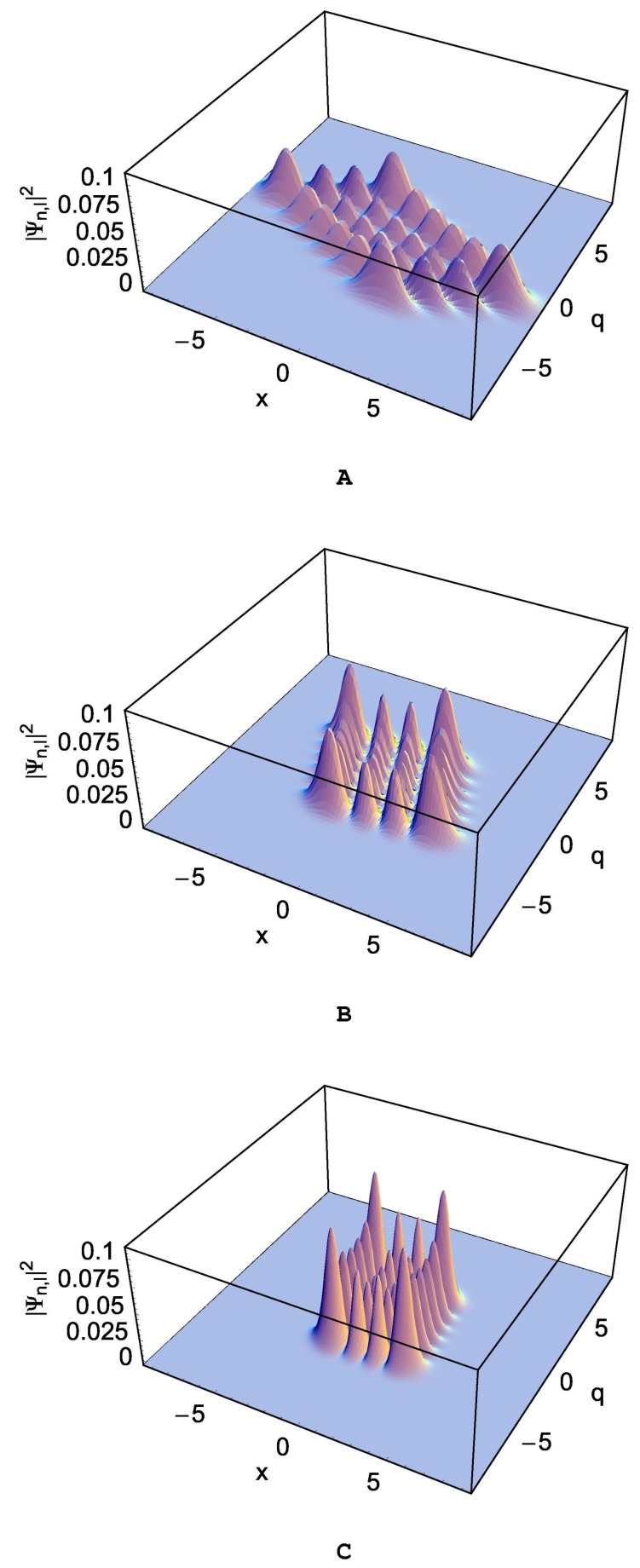
The probability density $|\Psi_{n,l}{\left(x,q,t\right)|}^{2}$ in the original system for several different values of ω with the choice of (*m*, *L*) = (0.5, 2.0) under the limit $\lambda \left(t\right)=\lambda_{0}$. This is associated to the wave functions given in Equation (19) with Equations (20), (21), (24), (25), (28), and (29). The values of (ω, Ω) are (0.5, 0.49) for (**A**), (1.0, 0.49) for (**B**), and (2.0, 0.49) for (**C**). We used (*n*, *l*) = (3, 5), ℏ=1, and $\lambda_{0}=0.1$.

Notice that the wave packets in the original system in the *x*-*p* coordinate have more or less been deformed depending on the values of parameters such as *m* and *L*, and rotated in proportion to θ from those in the transformed system. If θ is positive, the direction of rotation in the *x*-*p* plane is clockwise. The probability densities, $|\Psi_{n,l}{\left(x,q,t\right)|}^{2}$, are illustrated in Figure 2, Figure 3 and Figure 4. By comparing these figures with that in the transformed system represented in Figure 1, we can confirm the effects of the coupling on the behavior of the wave functions. Figure 2 is for several different values of ω with the choice of (*m*, *L*) = (1.0, 0.5) under the condition ω>Ω. The rotation angle θ can be evaluated from Equation (6) and is given by −0.76 rad for Figure 2A, −0.18 rad for Figure 2B, and −0.05 rad for Figure 2C.

We see from Figure 2 that the uncertainty of *x* is relatively small than that of *q*, which means that the wave packet is squeezed along the *x* coordinate. Such a squeezing effect becomes large as the difference ω−Ω increases. On the other hand, Figure 3, which is for the case that ω−Ω is negative, shows the squeeze of waves along the *q*-coordinate.

For the case of Figure 4 which is depicted under (*m*, *L*) = (0.5, 2.0), the wave packets exhibit *q*-squeezing. However, a weak *x*-squeezing for the waves also takes place as ω increases (see Figure 4C).

For the case that λ(t) is not a constant, it is necessary to use the quantum theory of time-dependent harmonic oscillators [9,23,24] in order to manage the Schrödinger equation, Equation (17) with Equation (12). According to that theory, the phases and the eigenfunctions in the transformed system are given in terms of time functions as [24]

(32) $$\begin{array}{ccc} \alpha_{n}\left(t\right) & = & -\left(n+1/2\right)\gamma_{1}\left(t\right), \end{array}$$

(33) $$\begin{array}{ccc} {\tilde{\alpha }}_{l}\left(t\right) & = & -\left(l+1/2\right)\gamma_{2}\left(t\right), \end{array}$$

(34) $$\begin{array}{ccc} \phi_{n}^{\prime }\left(x,t\right) & = & {\left(\frac{\sqrt{\kappa_{1}\left(t\right)/\pi }}{2^{n}n!}\right)}^{1/2}H_{n}\left(\sqrt{\kappa_{1}\left(t\right)}x\right)\exp\left[-\frac{\kappa_{1}\left(t\right)}{2}\left(1-\frac{i{\dot{s}}_{1}\left(t\right)}{{\dot{\gamma }}_{1}\left(t\right)s_{1}\left(t\right)}\right)x^{2}\right], \end{array}$$

(35) $$\begin{array}{ccc} {\tilde{\phi }}_{l}^{\prime }\left(q,t\right) & = & {\left(\frac{\sqrt{\kappa_{2}\left(t\right)/\pi }}{2^{l}l!}\right)}^{1/2}H_{l}\left(\sqrt{\kappa_{2}\left(t\right)}q\right)\exp\left[-\frac{\kappa_{2}\left(t\right)}{2}\left(1-\frac{i{\dot{s}}_{2}\left(t\right)}{{\dot{\gamma }}_{2}\left(t\right)s_{2}\left(t\right)}\right)q^{2}\right], \end{array}$$

where $\kappa_{i}=\mu {\dot{\gamma }}_{i}/\hbar$, while the time functions $s_{i}\left(t\right)$ and $\gamma_{i}\left(t\right)$ satisfy

(36) $$\begin{array}{c} {\ddot{s}}_{i}+w_{i}^{2}\left(t\right)s_{i}-C_{i}^{2}/s_{i}^{3}=0, \end{array}$$

(37) $$\begin{array}{c} {\dot{\gamma }}_{i}=C_{i}/s_{i}^{2}, \end{array}$$

where $C_{i}$ are arbitrary real constants.

From a minor evaluation through Equations (22) and (23), we have the wave solutions in the original system:

(38) $$\begin{array}{ccc} \phi_{n}\left(x,q,t\right) & = & {\left(\frac{\sqrt{\kappa_{1}\left(t\right)/\pi }}{2^{n}n!}\right)}^{1/2}H_{n}\left(\sqrt{\kappa_{1}\left(t\right)}Q_{1}\right)\exp\left[-\frac{\kappa_{1}\left(t\right)}{2}\left(1-\frac{i{\dot{s}}_{1}\left(t\right)}{{\dot{\gamma }}_{1}\left(t\right)s_{1}\left(t\right)}\right)Q_{1}^{2}\right], \end{array}$$

(39) $$\begin{array}{ccc} {\tilde{\phi }}_{l}\left(x,q,t\right) & = & {\left(\frac{\sqrt{\kappa_{2}\left(t\right)/\pi }}{2^{l}l!}\right)}^{1/2}H_{l}\left(\sqrt{\kappa_{2}\left(t\right)}Q_{2}\right)\exp\left[-\frac{\kappa_{2}\left(t\right)}{2}\left(1-\frac{i{\dot{s}}_{2}\left(t\right)}{{\dot{\gamma }}_{2}\left(t\right)s_{2}\left(t\right)}\right)Q_{2}^{2}\right], \end{array}$$

where $Q_{i}$ are given by Equations (30) and (31), but in terms of λ(t) which is not a constant.

The overall wave function is given by

(40) $$\Psi \left(x,q,t\right)=\sum_{n=0}^{\infty }\sum_{l=0}^{\infty }c_{n,l}\Psi_{n,l}\left(x,q,t\right),$$

where $c_{n,l}$ are complex numbers that yield $\sum_{n=0}^{\infty }\sum_{l=0}^{\infty }{\left|c_{n,l}\right|}^{2}=1$. Thus, we have obtained the full wave function of the system which is given by Equation (40) with Equations (19)–(21), (32), (33), (38), and (39). This wave function is crucial in unfolding quantum theory of the system and can be used to investigate diverse quantum characteristics that the system exhibits. We can use the wave function in estimating various quantum variables, such as energy eigenvalues, expectation values and fluctuations of the canonical variables, uncertainty products, Wigner distribution functions, and so on.

## 4. Conclusions

The quantum properties of the nanomechanical resonator coupled to a superconducting resonator via a small time-varying coupling constant have been investigated. We have used an adiabatic condition under the assumption that the time-variation of the coupling parameter is sufficiently slow. Due to the presence of the coupling term in the Hamiltonian, we cannot develop quantum theory for *x* and *q* coordinates independently in the original system. Hence, by means of the unitary transformation approach, we have decoupled *x* and *q* coordinates from each other in the expression of the Hamiltonian.

Through the transformation performed using a unitary operator, the Hamiltonian became a manageable one. Not only did the coupling term no longer appear in the transformed Hamiltonian, but the transformed system has also become much more simplified compared to the original one. More precisely speaking, the transformed system is composed of two independent harmonic oscillators with time-dependent angular frequencies. We accordingly have easily identified quantum solutions in the transformed system. By inverse transformation of those quantum solutions, we have obtained complete quantum solutions in the original system.

The exact wave functions derived here can be used for evaluating various quantum quantities of the coupled resonators, such as expectation values and fluctuations of the canonical variables, uncertainties, and energy eigenvalues, which are necessary for identifying the quantum characteristics of the system. It is also possible to derive Wigner distribution function of the system, which serves in describing signal processing, from the wave functions given in the text. Wigner distribution function can be used not only in estimating quantum corrections from classical statistical mechanics but also in demonstrating nonclassicalities of the system through quantum probability distribution. Terraneo et al. used Wigner distribution functions for the purpose of developing an efficient way for extracting information from the wave functions of quantum algorithms associated with quantum computation [25].

The probability densities which are the absolute square of the wave functions were illustrated in detail in the limit that λ is a constant. The wave packets in the *x*-*q* plane are rotated and deformed compared to those in the transformed system. The rotation direction is counterclockwise when ω−Ω is positive. Due to the coupling of the two sub-systems, the wave packets of the system underwent *x*-squeezing, *q*-squeezing, or both depending on the values of *m*, *L*, and the frequency difference ω−Ω. For the situation of *x*-squeezing (*q*-squeezing), the wave amplitude of the nanomechanical (the superconducting) resonator is small and, as a consequence, the nanomechanical (the superconducting) resonator has less energy. The energy of the nanomechanical resonator flows to the superconducting resonator, and vice versa [10,18]. From this process, the superconducting resonator extracts information about the nanomechanical resonator in order to control the nanoresonator.

The techniques for sideband cooling, frequency conversion, and state-swapping through coherent control of the resonator are important in quantum information systems, especially, in the realization of quantum computers. For the purpose of developing such techniques, the use of nanomechanical resonators is more effective than the use of nanophotonic resonators [11]. It is also noticeable that quantum data bus in quantum computers could be designed using nanomechanical resonators through their controllable coupling with qubits [2,26].

Our analysis regarding the wave functions of the system is useful for understanding the quantum behavior of the combined system. If we consider that theoretical analysis of a quantum system starts from the wave functions rigorously derived from the Schrödinger equation, the wave functions developed in this work are the basic tool for elucidating quantum properties of the system. Exact analyses of the quantum behaviors of nanoresonators based on such clarified quantum properties are necessary for realizing efficient quantum information processing.

The advantage of information processing and physical simulations using quantum computing devices rather than classical computers is that they make it possible to solve time-consuming problems within a polynomial time, such as factorizing large numbers which is classically very difficult to solve. The theoretical research for demonstrating the characteristics of quantum devices and their operations are important stepping stones for the development of quantum information science. While the system we treated here is a completely solvable one using our method, we also expect that quantum characteristics of more complicated namomecanical systems, including nanoresonators described by higher-order nonlinearity such as Duffing nonlinearity [27] and/or newly designed nanophotonic resonators [28,29], would be analyzed in the near future using the same method. Nanomechanical resonators are used for the detection of quantum states, spins, thermal fluctuations, etc., whereas nanophotonic resonators as passive optical components are mainly used for detecting light–matter interactions.

The coupled system investigated in this work can be used as a basic component of quantum computing systems. Quantum computation as a next generation technology should be developed together with other quantum information science such as quantum communication and quantum cryptography. Moreover recent development of neural computation through neural network with multi-core optical fibers [30,31] enhances the performance of the technology in quantum information science.

As a next task, the investigation of geometric phases that appear in the wave functions when parameters vary over time may be a good research topic which can be fulfilled on the basis of the quantum theory developed in the present work. Geometric phase can be applied not only to fundamental physics [23] but also to various next generation quantum technologies, such as quantum computing [32], interferometric imaging of microstructures [33], and beam steering in virtual/augmented reality displays [34].

## References

[1] Favero I., Marquardt F. (2014). Focus on optomechanics. New J. Phys..

[2] Rabl P., Kolkowitz S.J., Koppens F.H.L., Harris J.G.E., Zoller P., Lukin M.D. (2010). A quantum spin transducer based on nanoelectromechanical resonator arrays. Nat. Phys..

[3] Tetard L., Passian A., Venmar K.T., Lynch R.M., Voy B.H., Shekhawat G., Dravid V.P., Thundat T. (2008). Imaging nanoparticles in cells by nanomechanical holography. Nat. Nanotechnol..

[4] Sawadsky A., Kaufer H., Nia R.M., Tarabrin S.P., Khalili F.Y., Hammerer K., Schnabel R. (2015). Observation of generalized optomechanical coupling and cooling on cavity resonance. Phys. Rev. Lett..

[5] LaHaye M.D., Buu O., Camarota B., Schwab K.C. (2004). Approaching the quantum limit of a nanomechanical resonator. Science.

[6] Woolley M.J., Milburn G.J., Caves C.M. (2008). Nonlinear quantum metrology using coupled nanomechanical resonators. New J. Phys..

[7] Liao J.-Q., Law C.K., Kuang L.-M., Nori F. (2015). Enhancement of mechanical effects of single photons in modulated two-mode optomechanics. Phys. Rev. A.

[8] Wang D.-Y., Bai C.-H., Liu S., Zhang S., Wang H.-F. (2018). Optomechanical cooling beyond the quantum backaction limit with frequency modulation. Phys. Rev. A.

[9] Choi J.R. (2015). Hamiltonian dynamics and adiabatic invariants for time-dependent superconducting qubit-oscillators and resonators in quantum computing systems. Adv. Math. Phys..

[10] Tian L. (2009). Ground state cooling of a nanomechanical resonator via parametric linear coupling. Phys. Rev. B.

[11] Jacobs K., Nurdin H.I., Strauch F.W., James M. (2010). Frequency conversion: Side-band cooling, state-swapping, and coherent control of mechanical resonators. arXiv.

[12] Cleland A.N., Geller M.R. (2005). Mechanical quantum resonators. AIP Conf. Proc..

[13] Pechal M., Arrangoiz-Arriola P., Safavi-Naeini A.H. (2018). Superconducting circuit quantum computing with nanomechanical resonators as storage. Quantum Sci. Technol..

[14] Armour A.D., Blencowe M.P. (2008). Probing the quantum coherence of a nanomechanical resonator using a superconducting qubit: I. Echo scheme. New J. Phys..

[15] O’Connell A.D., Hofheinz M., Ansmann M., Bialczak R.C., Lenander M., Lucero E., Neeley M., Sank D., Wang H., Weides M. (2010). Quantum ground state and single-phonon control of a mechanical resonator. Nature.

[16] Teufel J.D., Donner T., Li D., Harlow J.W., Allman M.S., Cicak K., Sirois A.J., Whittaker J.D., Lehnert K.W., Simmonds R.W. (2011). Sideband cooling of micromechanical motion to the quantum ground state. Nature.

[17] Safavi-Naeini A.H., Chan J., Hill J.T., Alegre T.P.M., Krause A., Painter O. (2012). Observation of quantum motion of a nanomechanical resonator. Phys. Rev. Lett..

[18] Jacobs K., Nurdin H.I., Strauch F.W., James M. (2015). Comparing resolved-sideband cooling and measurement-based feedback cooling on an equal footing: Analytical results in the regime of ground-state cooling. Phys. Rev. A.

[19] Choi J.R. (2006). Exact solution of a quantized LC circuit coupled to a power source. Phys. Scr..

[20] Xiang Z.-L., Ashhab S., You J.Q., Nori F. (2013). Hybrid quantum circuits: Superconducting circuits interacting with other quantum systems. Rev. Mod. Phys..

[21] Peropadre B., Zueco D., Wulschner F., Deppe F., Marx A., Gross R., García-Ripoll J.J. (2013). Tunable coupling engineering between superconducting resonators: From sidebands to effective gauge fields. Phys. Rev. B.

[22] Fedortchenko S., Felicetti S., Marković D., Jezouin S., Keller A., Coudreau T., Huard B., Milman P. (2017). Quantum simulation of ultrastrongly coupled bosonic modes using superconducting circuits. Phys. Rev. A.

[23] Lakehal H., Maamache M., Choi J.R. (2016). Novel quantum description for nonadiabatic evolution of light wave propagation in time-dependent linear media. Sci. Rep..

[24] Khandekar D.C., Lawande S.V. (1975). Exact propagator for a time-dependent harmonic oscillator with and without a singular perturbation. J. Math. Phys..

[25] Terraneo M., Georgeot B., Shepelyansky D.L. (2005). Quantum computation and analysis of Wigner and Husimi functions: Toward a quantum image treatment. Phys. Rev. E.

[26] Xue F., Wang Y.D., Sun C.P., Okamoto H., Yamaguchi H., Semba K. (2007). Controllable coupling between flux qubit and nanomechanical resonator by magnetic field. New J. Phys..

[27] Lü X.-Y., Liao J.-Q., Tian L., Nori F. (2015). Steady-state mechanical squeezing in an optomechanical system via Duffing nonlinearity. Phys. Rev. A.

[28] Malka D., Cohen M., Turkiewicz J., Zalevsky Z. (2015). Optical micro-multi-racetrack resonator filter based on SOI waveguides. Photonics Nanostruct. Fundam. Appl..

[29] McGehee W.R., Michels T., Aksyuk V., McClelland J.J. (2017). Two-dimensional imaging and modification of nanophotonic resonator modes using a focused ion beam. Optica.

[30] Cohen E., Malka D., Shemer A., Shahmoon A., Zalevsky Z., London M. (2016). Neural networks within multi-core optic fibers. Sci. Rep..

[31] Shabairou N., Cohen E., Wagner O., Malka D., Zalevsky Z. (2018). Color image identification and reconstruction using artificial neural networks on multimode fiber images: Towards an all-optical design. Opt. Lett..

[32] Liu T., Cao X.-Z., Su Q.-P., Xiong S.-J., Yang C.-P. (2016). Multi-target-qubit unconventional geometric phase gate in a multi-cavity system. Sci. Rep..

[33] Tavrov A.V., Miyamoto Y., Kawabata T., Takeda M., Andreev V.A. (2000). Interferometric microimaging based on geometrical spin-redirection phase. Opt. Lett..

[34] Lee Y.-H., Tan G., Zhan T., Weng Y., Liu G., Gou F., Peng F., Tabiryan N.V., Gauza S., Wu S.-T. (2017). Recent progress in Pancharatnam-Berry phase optical elements and the applications for virtual/augmented realities. Opt. Data Process. Storage.

